# How many more polymorphs of ROY remain undiscovered†

**DOI:** 10.1039/d1sc06074k

**Published:** 2021-12-13

**Authors:** Gregory J. O. Beran, Isaac J. Sugden, Chandler Greenwell, David H. Bowskill, Constantinos C. Pantelides, Claire S. Adjiman

**Affiliations:** Department of Chemistry, University of California Riverside Riverside CA 92521 USA gregory.beran@ucr.edu; Department of Chemical Engineering, Sargent Centre for Process Systems Engineering, Imperial College London London SW7 2AZ UK

## Abstract

With 12 crystal forms, 5-methyl-2-[(2-nitrophenyl)amino]-3-thiophenecabonitrile (a.k.a. ROY) holds the current record for the largest number of fully characterized organic crystal polymorphs. Four of these polymorph structures have been reported since 2019, raising the question of how many more ROY polymorphs await future discovery. Employing crystal structure prediction and accurate energy rankings derived from conformational energy-corrected density functional theory, this study presents the first crystal energy landscape for ROY that agrees well with experiment. The lattice energies suggest that the seven most stable ROY polymorphs (and nine of the twelve lowest-energy forms) on the Z′ = 1 landscape have already been discovered experimentally. Discovering any new polymorphs at ambient pressure will likely require specialized crystallization techniques capable of trapping metastable forms. At pressures above 10 GPa, however, a new crystal form is predicted to become enthalpically more stable than all known polymorphs, suggesting that further high-pressure experiments on ROY may be warranted. This work highlights the value of high-accuracy crystal structure prediction for solid-form screening and demonstrates how pragmatic conformational energy corrections can overcome the limitations of conventional density functionals for conformational polymorphs.

## Abstract 2

Crystal structure prediction suggests that the low-energy polymorphs of ROY have already been found, but a new high-pressure form is predicted.

## Graphical abstract

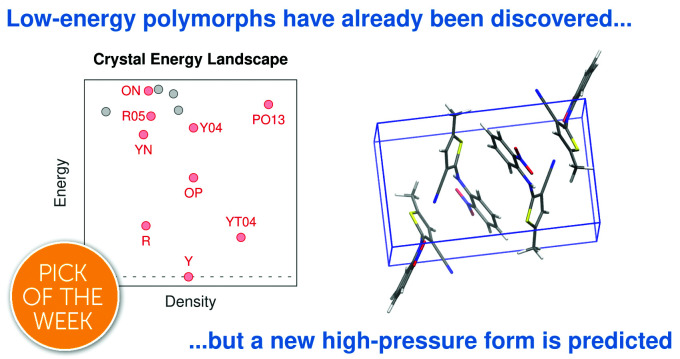

## 1 Introduction

Estimates indicate that about half of organic molecules exhibit polymorphism in the solid state,^1^ and these changes in crystal packing can profoundly alter physical properties such as color, stability, solubility, and carrier mobility. While the discovery of two or three polymorphs for a given species is common, some molecules are prolific polymorph formers that can adopt many more crystal forms. For example, ten fully characterized polymorphs have been reported for anti-cancer drug candidate galunisertib,^2^ nine for flufenamic acid,^3^ aripiprazole,^4^ and tolfenamic acid,^5^ six for the energetic material triacetone-triperoxide (TATP),^6^ and at least five polymorphs for many other species.^7–14^ Large numbers of crystalline phases are also found for small molecules such as water,^15^ nitrogen,^16^ and carbon dioxide.^17,18^

**Fig. 1:**
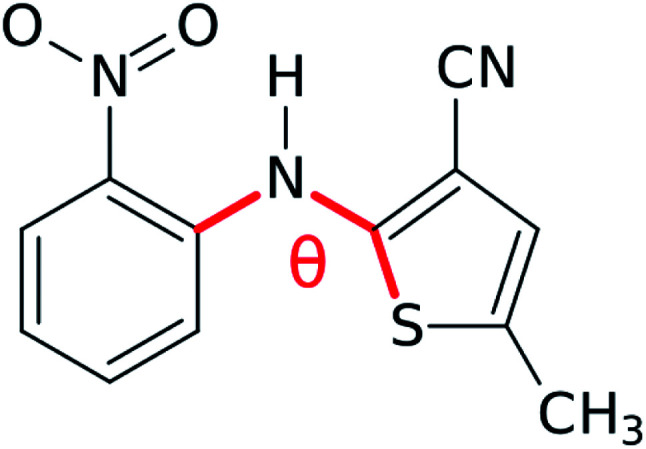
The ROY molecule. Its different conformational polymorphs and their associated colors generally involve changes in the S–C–N–C dihedral angle *θ*.

The molecule 5-methyl-2-[(2-nitrophenyl)amino]-3-thiophenecabonitrile in Fig. 1, which is often called ROY due to its vividly-colored red, orange, and yellow crystals, is currently the most prolific organic polymorph former known. Twelve fully characterized ROY crystal structures have been discovered in the past twenty-five years: Y,^19,20^ ON,^19,20^ R,^19,20^ OP,^20^ YN,^20^ ORP,^20^ YT04,^21^ Y04,^21,22^ R05,^23,24^ PO13,^25,26^ R18,^27^ and Y19.^26^ The structure for a thirteenth RPL polymorph^28^ has been proposed,^29^ but it has not yet been definitively characterized. Structures for four of these forms (PO13, R18, Y19, and Y04) have been reported only since 2019. Whereas Y04 and PO13 were previously known but incompletely characterized, R18 and Y19 are entirely new polymorphs. This rapid increase in the number of solved ROY polymorphs seems to support Walter McCrone's oft-cited conjecture^30^ that “in general, the number of forms known for a given compound is proportional to the time and money spent in research on that compound.” It also raises the question: how many additional polymorphs of ROY remain to be discovered?

Computational crystal structure prediction (CSP) is increasingly used to survey the landscape of potential crystal structures as a complement to experimental solid form screening efforts.^31,32^ In the pharmaceutical industry, for example, CSP has recently contributed to the discovery and structural determination of new polymorphs of species such as dalcetrapib,^33^ aspirin,^34^ paracetamol,^35^ β-estradiol,^36^ galunisertib,^2^ and olanzapine.^37^ It has similarly helped guide the discovery of new, highly porous organic molecular crystals.^38,39^

**Fig. 2:**
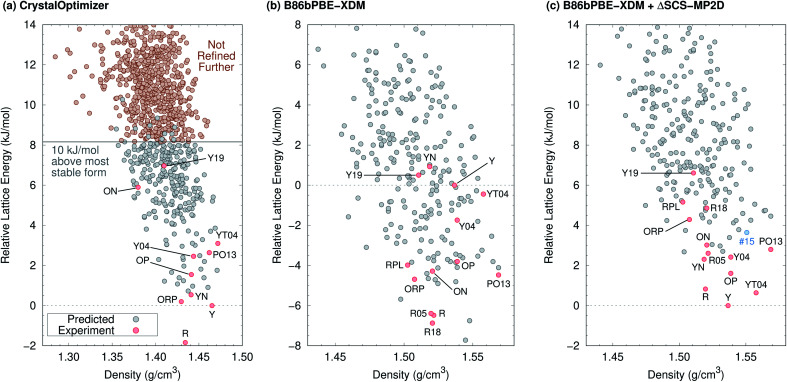
Predicted crystal energy landscapes (a) from CrystalOptimizer with the initial force field, (b) after B86bPBE-XDM geometry relaxation and energy refinement of approximately 300 low-energy structures (red and gray circles), and (c) after single-point SCS-MP2D monomer conformational energy corrections to the B86bPBE-XDM landscape. Experimentally observed structures are indicated in red, while the potential high-pressure candidate structure #15 is indicated in blue. DFT-optimized structures and energies are provided in ESI.†

The 2012 ROY CSP landscape of Vasileiadis *et al.*^40^ includes all ten known ROY crystal polymorphs whose structures contain a single molecule in the asymmetric unit (Z′ = 1). However, these experimental structures lie as high as rank 144 (Y19) on that landscape.^26^ In the initial new Z′ = 1 landscape generated here (Fig. 2a) that will be discussed in detail below, the Y19 polymorph rises further to rank 172. At face value, these crystal energy landscapes appear to suggest that large numbers of ROY polymorphs are possible and that many potential low-energy forms have not yet been discovered.

The fact that CSP routinely predicts far more seemingly viable candidate structures than are known experimentally has been the subject of much discussion.^39,41–43^ Multiple reasons have been advanced to account for this discrepancy, including inadequacies in the computational models, molecular rearrangements that occur during the crystal nucleation and growth process, metastability of the predicted structures, and/or a failure to perform the correct crystallization experiment. While experimental explanations may sometimes be appropriate, questions regarding the accuracy of the computational model(s) always lurk behind predicted crystal energy landscapes. Those model inadequacies can stem from limitations in the lattice energy models, neglect or approximation of the phonon contributions and free energies, and/or failing to account for the dynamical motions of the molecules which can merge multiple lattice energy minima into a single free energy basin at finite temperature.

Focusing on the quality of lattice energy models, substantial progress in CSP has been made thanks to the widespread use of van der Waals-inclusive density functional theory (DFT) models^44–47^ to refine the crystal energy landscapes.^48,49^ It has led to many successful predictions in the blind tests^50–52^ and other applications.^49,53–67^ DFT has largely replaced traditional force field models for determining the final crystal structure rankings, for example. However, common density functionals predict the relative energetics of the ROY polymorphs poorly,^24,29,68^ significantly over-stabilizing the red and orange polymorphs while frequently predicting the thermodynamically most stable Y polymorph to be among the least stable forms. This polymorph energy ranking problem stems from delocalization error^69^ in the approximate density functionals which artificially stabilizes crystal structures containing more planar conformations of ROY that exhibit greater π conjugation.^29,55,70,71^ Delocalization error also can cause over-binding of halogen-bonded crystals^72^ and even spurious proton transfer in acid–base co-crystals due to artificial stabilization of salt forms over the neutral co-crystal.^73^ We previously demonstrated that the stability rankings of the major experimentally-known polymorphs in ROY and a number of other systems improve dramatically if one corrects the intramolecular conformational energies using an electronic structure model that does not suffer from delocalization error.^71,74,75^

Whereas the ROY studies examining the impact of delocalization error described above considered only experimentally-known polymorphs, the present study now performs crystal structure prediction and applies our conformational energy correction approach to investigate the landscape containing all experimentally-known polymorphs of ROY and hundreds of additional candidate structures. This effort results in the first ROY crystal energy landscape that is highly consistent with experiment. It suggests that researchers have already discovered most if not all of the highly stable ROY polymorphs. Realizing any new polymorphs at ambient conditions will likely require selective crystallization strategies capable of kinetically trapping more metastable structures. On the other hand, one currently unknown candidate structure is predicted to become thermodynamically more stable than all existing ROY polymorphs at around 10 GPa of pressure, suggesting that high pressure experiments might discover a new polymorph.

## 2 Theoretical approach

Candidate crystal structures of ROY were generated *via* crystal structure prediction, as described briefly below. See ESI† for further details. The initial stages of the CSP protocol follow the general strategy from Pantelides *et al.*^76^ and were employed in a 2012 study of ROY.^40^

First, relevant ROY conformations and local approximate models^77^ describing the intramolecular conformational energetics for two key conformational degrees of freedom were generated *via* gas-phase studies at the B3LYP/6-31G(d,p) level of theory using Gaussian 09.^78^ See ESI† for details. Second, a global CSP search for low-energy minima on the Z′ = 1 lattice energy surface was performed using CrystalPredictor II,^79^ using the smooth intramolecular potential algorithm^80^ for intramolecular interactions and parameters from the FIT potential^81–84^ set for intermolecular interactions. Two million minimizations in 61 space groups were carried out. After removal of duplicates, 2869 distinct crystal structures were identified within 20 kJ mol^−1^ of the global minimum. Third, the lowest 1000 of these structures were locally minimized with additional conformational flexibility in CrystalOptimizer,^85^ using B3LYP/6-31G(d,p) to describe the intramolecular energetics, multipolar electrostatics (up to hexadecapoles),^86^ and repulsion/dispersion parameters from the FIT potential set.

Fourth, all structures within 10 kJ mol^−1^ of the global minimum energy structure were fully relaxed with dispersion-corrected periodic planewave DFT calculations using the B86bPBE density functional^87,88^ and the exchange-dipole moment (XDM) dispersion correction^89^ in Quantum Espresso.^90,91^ To identify any additional higher-lying structures from the CrystalOptimizer landscape that might also be important, the DFT-optimized crystal geometries and energies from the 50-lowest-energy CrystalOptimizer structures were used to refit the repulsion/dispersion parameters with CrystalEstimator.^92^ Such molecule-specific tailoring has been shown to lead to improved outcomes in CSP.^48,93–99^ After refining all 1000 structures from stage 3 with the modified potential, around a dozen additional structures fell within the 10 kJ mol^−1^ window and were also relaxed with DFT. Because the search only examined Z′ = 1, the three experimentally-reported Z′ = 2 polymorphs (R05, R18, and RPL) were added to the set manually. For the RPL polymorph, the proposed structure from ref. 29 was used. In total, approximately 300 crystal structures were relaxed with B86bPBE-XDM. After removal of duplicates, the landscape includes 264 crystal structures.

Finally, because delocalization error in generalized gradient approximation (GGA) and hybrid functionals artificially stabilizes more planar conformations of ROY,^29,70,71^ a single-point gas-phase monomer conformational energy correction was applied to each crystal.^71^ These corrections replace the B86bPBE-XDM intramolecular conformational energy with one computed from spin-component-scaled dispersion-corrected second-order Møller–Plesset perturbation theory (SCS-MP2D) at the complete-basis-set limit.^100^ This model reproduces benchmark coupled cluster theory conformational energies of ROY well (Fig. S3†) at modest computational cost. The conformationally-corrected energy per unit cell is computed as:1
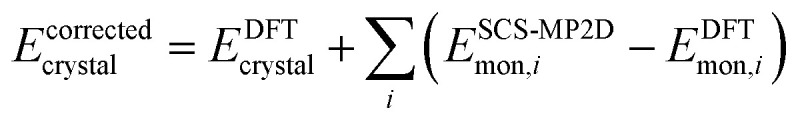
where *E*_mon,*i*_ is the gas-phase energy of the *i*-th monomer in the unit cell (in its crystalline geometry) and *i* runs over all molecules in the cell. Space group symmetry can be exploited to compute corrections only for the symmetrically unique monomers. For ROY, the single-point monomer correction performed here requires 1–2 orders of magnitude less computational effort than the periodic DFT geometry optimization, depending on the unit cell size. SCS-MP2D calculations were performed using PSI4 and a custom implementation^101^ of the spin-component scaling and dispersion correction.

## 3 Results and discussion

### 3.1 The ROY crystal energy landscape

**Fig. 3:**
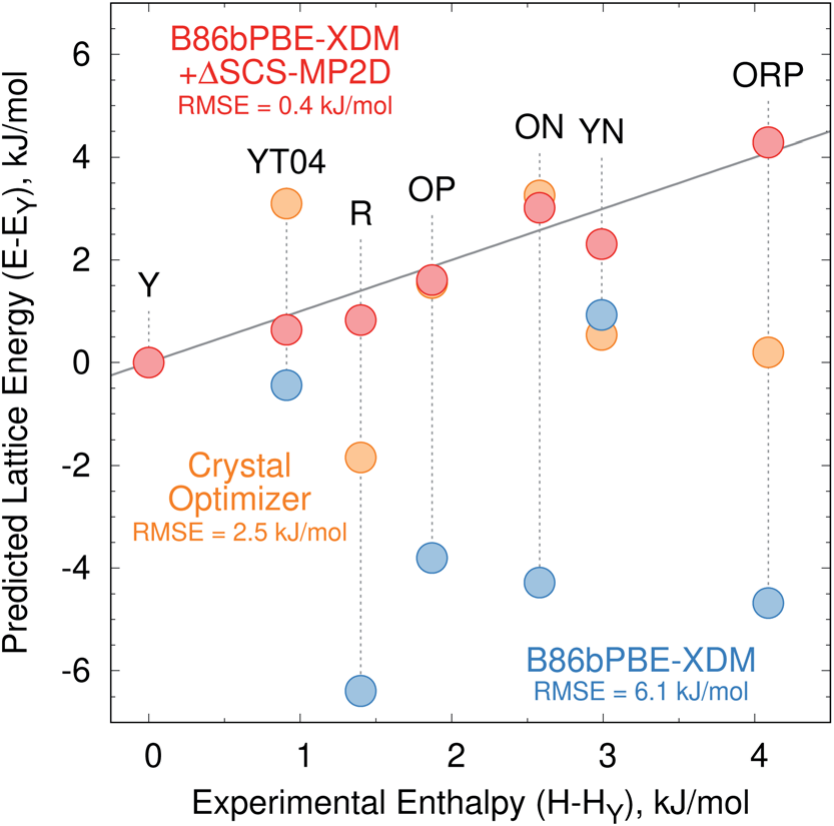
Relative lattice energies for seven ROY polymorphs predicted with the CrystalOptimizer potential (orange), B86bPBE-XDM (blue) and B86bPBE-XDM + the SCS-MP2D monomer-correction (red) compared against the experimentally measured relative enthalpies (kJ mol^−1^). Points lying along the solid gray line would represent perfect agreement between the relative theoretical lattice energies and experimental enthalpies.

Fig. 2a presents the initial CrystalOptimizer energy landscape from stage 3 of the CSP protocol. This *Z*′ = 1 landscape is densely populated, containing 284 structures within 10 kJ mol^−1^ of the R polymorph which it predicts to be the global minimum energy structure. Similar to an earlier version of this landscape,^40^ it includes all ten experimentally-known *Z*′ = 1 polymorphs. However, the energy model incorrectly predicts the R polymorph to be 1.8 kJ mol^−1^ more stable than form Y. Moreover, while many of the experimental polymorphs appear among the 30 lowest structures, forms ON (rank 115) and Y19 (rank 172) lie considerably higher. As shown in Fig. 3, the relative CrystalOptimizer lattice energies correlate moderately well with the relative experimental enthalpies that Yu reported for the Y, YT04, R, OP, ON, YN, and ORP polymorphs,^102^ with a root-mean-square error (RMSE) of 2.5 kJ mol^−1^. The largest errors of 3–4 kJ mol^−1^ occur for the R and ORP polymorphs. While perfect agreement between relative 0 K lattice energies and relative finite-temperature enthalpies cannot be expected due to differences in the enthalpy phonon contributions between forms that are absent in the lattice energies, this data suggests that the CrystalOptimizer energy rankings probably exhibit appreciable errors relative to the magnitude of the energy differences between ROY polymorphs.

Next, all structures indicated in gray or red in Fig. 2a were fully relaxed with periodic density functional theory using the dispersion-corrected B86bPBE-XDM density functional. This set includes all 284 structures lying within 10 kJ mol^−1^ of form R on the CrystalOptimizer landscape plus a handful of higher-energy structures that were stabilized upon refitting of the CrystalOptimizer potential, as described in the Theoretical methods section.

In the end, after relaxing approximately 300 crystal structures with DFT and removing duplicates, the landscape contains 264 crystal structures. DFT refinement broadens the energy range of these structures considerably to 22 kJ mol^−1^, and Fig. 2b plots the lowest 16 kJ mol^−1^ region of this landscape. The DFT-optimized structures are also noticeably more dense compared to the CrystalOptimizer ones, reflecting the differences between an intermolecular potential fitted to finite-temperature crystal structures and 0 K electronic structure calculations. Nevertheless, the DFT-optimized structures exhibit good agreement with their experimental counterparts, with an average 15-molecule cluster root-mean-square deviation (RMSD15)^103^ value of 0.23 ± 0.08 Å (Table S2†).

Unfortunately, the B86bPBE-XDM energy rankings agree poorly with experiment, similar to what has been found in previous DFT studies of ROY.^24,29,68,70,71^ As shown in Fig. 3, the B86bPBE-XDM lattice energies exhibit an RMS error of 6.1 kJ mol^−1^ compared to the relative experimental enthalpies, more than double the error of the CrystalOptimizer potential. Most notably, the red- and orange-colored experimental polymorphs are substantially overstabilized relative to the yellow ones. B86bPBE-XDM predicts 92 structures to be more stable than the experimentally most stable form Y, and 96% of those lower-energy structures adopt intramolecular conformations associated with red/orange polymorphs instead of yellow ones. Forms R18, R, and R05 are predicted to be among the most stable structures, and an experimentally-unknown structure is predicted to be the global minimum.

The poor B86bPBE-XDM energy rankings reflect the impact of delocalization error in the density functional, which artificially stabilizes structures with more planar ROY conformations.^29,68,70,71^ In contrast, the CrystalOptimizer energy model derives its intramolecular conformational energies from the hybrid B3LYP functional, which is less impacted by delocalization error than the GGA functional B86bPBE-XDM. Nevertheless, B3LYP and other hybrid functionals do still overstabilize the red and orange conformations, albeit to a lesser extent.^71^

To address the delocalization error, the intramolecular DFT conformational energies were replaced with wavefunction-based SCS-MP2D ones that do not suffer from delocalization error. This computationally inexpensive correction (*cf.*eqn (1)) dramatically improves the crystal energy landscape (Fig. 2c). Form Y becomes the most stable polymorph, followed by YT04, R, OP, YN, Y04, R05, PO13, ON, ORP, R18, RPL, and Y19. As shown in Fig. 3, these conformational energy-corrected relative lattice energies correlate excellently with the relative experimental enthalpies, reducing the rms error from 6.1 kJ mol^−1^ to only 0.4 kJ mol^−1^. This represents a remarkable improvement, despite the aforementioned caveat about comparing electronic lattice energies and experimental enthalpies. Moreover, the predicted polymorph stability ordering for these seven forms matches the experimental one almost perfectly. Only the YN and ON energy ordering is reversed, though the predicted energy difference is only 0.7 kJ mol^−1^.^71^ This excellent agreement between the predicted relative lattice energies and relative experimental enthalpies supports the assumption of the monomer correction approach that the B86bPBE-XDM functional is describing the intermolecular interactions well in this system.

The predicted relative stabilities are also consistent with available experimental evidence beyond that shown in Fig. 3. For example, the model predicts Y04 to be less stable than YT04 and R, which is consistent with the experimentally observed spontaneous conversion of Y04 to those forms.^21,22^ Similarly, forms R05 and YN lie above Y and R, which is consistent with experimentally observed transformations over time.^20,24^

The ORP, R18, RPL, and Y19 polymorphs lie 1–4 kJ mol^−1^ above the other experimentally known polymorphs in Fig. 2c, but this relative instability also seems consistent with available experimental information. For example, ORP is metastable and converts to Y over time,^20^ and the relative lattice energy agrees well with the experimental enthalpy difference (Fig. 3). RPL is also highly metastable experimentally, converting to lower-energy form YN in a matter of hours or days, and even faster upon even slight mechanical pressure.^28^ The stabilities of the newly discovered R18 and Y19 forms have not been reported, but unconventional crystallization techniques were required to produce each one: R18 was discovered during extensive screening *via* encapsulated nanodroplet crystallization.^27^ Y19 was crystallized using seeds of a mixed crystal consisting of a 40 : 60 mixture of ROY and FuROY, the latter of which replaces the sulfur atom with an oxygen atom.^26^ In other words, the relative instability predicted for these forms is plausible given the difficulties in crystallizing them experimentally.

### 3.2 Potential for discovering new polymorphs

Having obtained a CSP landscape that reproduces important features of the experimentally known polymorphs, the potential for discovering new polymorphs in the future can be assessed. The crystal energy landscape contains 174 crystal structures within the commonly studied 10 kJ mol^−1^ lattice energy window, 161 of which have not been observed experimentally. 59 of those unknown structures lie in the 6.6 kJ mol^−1^ energy window between the most stable (Y, rank #1) and least stable (Y19, rank #72) experimental forms.

Most significantly, the seven lowest-energy structures on the CSP landscape have already been discovered experimentally: Y, YT04, R, OP, YN, Y04, R05. The rank #10 (PO13) and #12 (ON) structures are also already known. Only 13 currently undiscovered structures lie below the metastable and/or difficult-to-crystallize energy window of forms ORP, R18, RPL, and Y19.

The most stable of those candidates occur at ranks #8, #9, #11, and #13, all lying within about 3 kJ mol^−1^ of form Y. Structures #8, #9 and #11 would be yellow polymorphs. The first two adopt an intramolecular conformation almost identical to that of YT04, while the third nearly matches the Y polymorph conformation. With a conformation similar to forms ON and OP, structure #13 would probably be orange. The intermolecular packing arrangements in #8, #11, and #13 differ from any of the experimentally-known polymorphs. In contrast, structure #9 adopts the same 1-D wine-rack motif as YT04, but with different packing relationships between the 1-D stacks. Given the common packing motif, crystallization might reasonably lead preferentially to the formation of the thermodynamically more stable YT04 polymorph over structure #9. Several of the other lower-energy candidate structures also exhibit varying degrees of packing motif similarity with known forms that could make them difficult to crystallize instead of the already known polymorphs. Finally, we note that the two predicted structures that were exceptionally stable on the DFT landscape in Fig. 2b are destabilized considerably by applying the conformational correction, increasing to ranks #17 (+3.8 kJ mol^−1^) and #43 (+5.5 kJ mol^−1^).

Crystal packing similarities between the candidate structures and the polymorphs of FuROY, the ROY-analog which replaces the sulfur with an oxygen, were also investigated. The only notable partial packing similarity occurs between the orange-red polymorph of FuROY and the rank #194 structure. However, at 10.6 kJ mol^−1^ less stable than form Y, structure #194 seems unlikely to be realized experimentally. The lack of low-energy structures with strong packing similarities to the FuROY polymorphs is consistent with the failure of FuROY homoseeding to produce any new polymorphs of ROY.^26^

What about crystal structures with more than one molecule in the asymmetric unit (*Z*′ > 1)? While the present crystal structure prediction searched over only *Z*′ = 1 structures, the 2019 study by Nyman *et al.* also included *Z*′ = 2 structures.^29^ That crystal landscape is imperfect: it found only 6 of the then 9 known experimental polymorphs, and its structures are heavily biased towards more planar ROY conformations (*i.e.* red/orange-colored polymorphs) due to delocalization error in the density functional used to produce it. Nevertheless, it can provide insight into the potential for important *Z*′ = 2 structures not considered in the current search. As discussed in Section S2.4,† a single-point energy re-ranking with B86bPBE-XDM and the SCS-MP2D conformational energy correction of the 500 lowest-energy structures on that landscape found one experimentally-unknown candidate within 3 kJ mol^−1^ of form Y (*i.e.* the energy window containing the nine most stable experimental polymorphs in Fig. 2c). However, this structure exhibits significant packing similarities to the more stable YN polymorph, suggesting it might be difficult to crystallize experimentally. The landscape also contains nearly 50 unknown *Z*′ = 2 structures within 5 kJ mol^−1^ of form Y. Similar to what was found for the landscape in Fig. 2c, however, it is unclear whether any of those structures are particularly likely to be realizable experimentally.

So while the conformational energy-corrected crystal energy landscapes contain many unknown crystal structures that might conceivably be crystallized experimentally, it seems likely that doing so would prove difficult for the vast majority of the candidate structures in Fig. 2c. The lowest-energy forms have already been discovered. Most of the experimentally unknown predicted structures lie higher in energy than the already difficult-to-produce metastable forms like RPL, R18, and Y19, while several of the lower-energy candidates exhibit partial packing similarities to more experimental polymorphs. Of course, one should never entirely rule out the potential for experimental ingenuity and/or serendipity to discover some of these hypothetical forms.

### 3.3 ROY polymorphs at high pressure

Given the dearth of promising new low-energy candidate structures to be crystallized at ambient conditions, we turn our attention to the potential for new high-pressure polymorphs of ROY. Experimental studies have investigated the behavior of the Y,^104^ OP,^105^ and ON^106^ polymorphs at pressures up to 5.2 GPa, 9.3 GPa, and 4.2 GPa, respectively. With the exception of form ON,^106^ hydrostatic pressure generally flattens the key SCNC intramolecular dihedral angle between the two aromatic rings. In form Y, this dihedral flattening results in a “collapsable wine rack” compression of the crystal structure and an associated piezochromic color change from yellow to red.^104^ However, no pressure-induced polymorphic phase transitions have been yet been observed in ROY.

Given the number of high-density forms with relatively low lattice energies, we investigate the pressure-dependent relative enthalpies for the 12 fully characterized experimental forms and 14 additional structures exhibiting packing densities similar to or greater than form Y and energies less than 6 kJ mol^−1^ above Y at ambient pressure. See Fig. S5† for the complete set of structures considered. The low-density, high-energy RPL polymorph was omitted here because it is unlikely to be energetically relevant at higher pressures, and its large *Z*′ = 16 unit cell makes it considerably more computationally expensive to model than the other forms. For computational efficiency in screening these 26 crystal structures, the enthalpy is approximated by H = *E*_latt_ + PV, neglecting phonon and/or finite-temperature contributions.

At zero pressure, the most stable form Y is more dense than most of the other experimental forms (Fig. 2c). Applying external pressure further destabilizes the less dense polymorphs such as R, YN, R05, ON, *etc.* While forms OP and Y04 are slightly more dense than form Y at ambient pressure, they prove less compressible. Pressures up to ∼1 GPa stabilize these two forms relative to Y, but they are then destabilized as the pressure increases further. Only the most dense YT04 and PO13 polymorphs are consistently stabilized relative to form Y by increasing pressure. Both polymorphs are enthalpically more stable than Y already at 1 GPa, and YT04 is preferred over Y and PO13 up to at least 15 GPa.

**Fig. 4:**
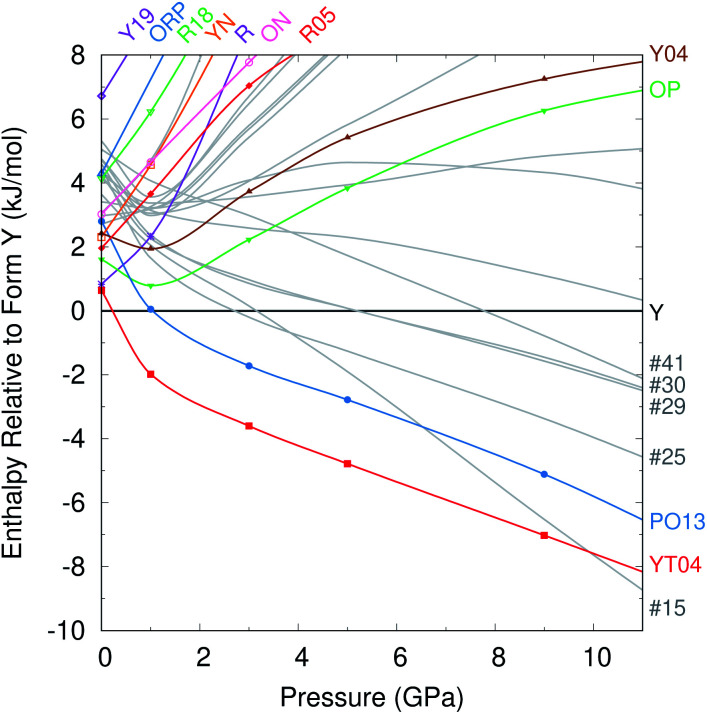
Enthalpies of the different crystal structures relative to form Y as a function of pressure, in kJ mol^−1^.

Most of the 14 hypothetical CSP candidate structures examined are similarly destabilized at high pressures. However, five of these candidates become more stable than form Y by ∼8 GPa. Most notably, structure #15 is 1.4% more dense than form Y at ambient conditions and slightly more compressible. So while structure #15 lies 3.6 kJ mol^−1^ above form Y without external pressure, it is predicted to become more stable than form Y by 3 GPa, and more stable than all experimentally known forms by ∼10 GPa. In contrast, the other four candidate structures stabilized by pressure appear likely to remain less stable than forms PO13 and YT04 even at pressures somewhat beyond 10 GPa (Fig. 4).

**Fig. 5:**
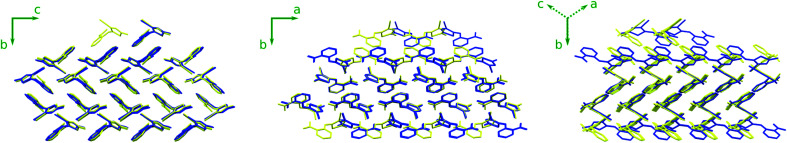
Comparison of form Y (yellow) and structure #15 (blue) at ambient pressure. Cell axes are indicated for form Y. For the middle and right perspectives in particular, note how the structures share a common packing motif in the central layers but the adjacent layers above and below in the *b* direction differ by mirror image inversion about the *a* axis.

Like form Y, structure #15 would exhibit piezochromism. At ambient pressure, it should be yellow with a 118° SCNC dihedral angle (similar to forms Y, YN, and YT04). However, by 10 GPa, the dihedral angle is predicted to flatten to a PO13-like 131°, thereby shifting the crystal color toward orange or red. The intermolecular packing of structure #15 also shares similarities with form Y (Fig. 5). It exhibits the same herringbone-layer of π-stacked 6-membered rings as form Y, but the adjacent layers differ by inversion of the hydrogen-bonding partners. This high structural similarity could favor the crystallization of the more stable form Y over structure #15 at ambient conditions.

On the other hand, these structural similarities might also suggest that form Y could transform to structure #15 at high pressures. Previous high-pressure experiments did not discover any new high pressure polymorphs. However, they compressed form Y only to 5.2 GPa.^104^ While structure #15 should be more stable already in that regime, perhaps the stability difference was insufficient to overcome the kinetic barrier for the solid–solid phase transition. A second study compressed the OP polymorph up to 9.2 GPa,^105^ at which point it is far less stable than many other predicted and experimental polymorphs. However, the crystal packing in form OP differs considerably from that found in any of these lower-energy structures, which might have created an insurmountable kinetic barrier to phase transition. Based on the predicted enthalpy differences and the crystal packing similarities with form Y, perhaps pressurizing form Y to near ∼10 GPa might enable the experimental formation of structure #15. Alternatively, if the kinetic barriers to solid–solid transformation from form Y to #15 are too high, high-pressure liquid-phase recrystallizations might also provide a route to forming structure #15.

### 3.4 Polymorph color analysis

Color polymorphism can arise from changes in intramolecular conformation or intermolecular packing.^107^ Because the color polymorphism in ROY stems primarily (but not exclusively) from the intramolecular conformation,^106,108^ we examine the distribution of conformations found on the CSP landscape. In the gas-phase, the ROY conformational energy profile exhibits two basins, separated by a barrier for SCNC dihedral angle around 80°. However, the polymorph color is largely driven by the extent of π conjugation resulting from the conformation, with yellow forms adopting dihedral angles of ∼90 ± 40° and red/orange ones adopting more planar dihedral angles below ∼50° or above ∼130°.

**Fig. 6:**
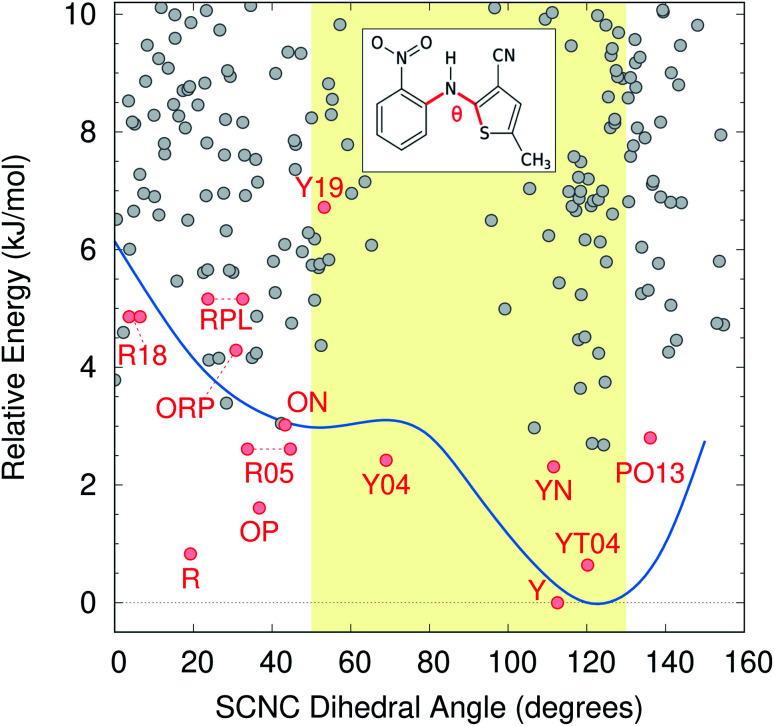
ROY crystal structure energy landscape as a function of the intramolecular conformation, as defined by the key SCNC dihedral angle. The gas-phase SCS-MP2D conformational energy scan over this dihedral angle is superimposed on the landscape in blue, while the yellow shaded region indicates the approximate dihedral angle range that leads to yellow polymorphs instead of red/orange ones.

As shown in Fig. 6, neither conformational energy basin is particularly preferred among the candidate structures—both basins contain similar numbers of structures within the 10 kJ mol^−1^ energy window. The modest conformational strain associated with dihedral angles less than ∼80° is apparently readily overcome through improved intermolecular packing. Such balanced interplay between intramolecular conformation and intermolecular packing forces is frequently what drives conformational polymorphism.^109^ Nevertheless, Fig. 6 reveals that conformations typically associated with red/orange polymorphs are about twice as likely on the landscape as are those associated with yellow crystals, which essentially matches the proportions found among the current experimental polymorphs.

## 4 Conclusions

This study has produced the first crystal structure prediction landscape for ROY that is consistent with available experimental evidence. This was achieved by augmenting the CSP approach that had previously produced the most realistic landscape with conformational energy corrections to DFT lattice energies to overcome the limitations of delocalization error in the approximate density functionals. The relative lattice energies of the known polymorphs agree well with relative experimental enthalpies and other qualitative observations about polymorph stabilities. In the future, one might investigate the accuracy of the DFT intermolecular description further, though the good agreement with experiment provides some confidence that those interactions are described well in the model used here. While the landscape presented here appears accurate, future work could improve it further by examining structures with multiple molecules in the asymmetric unit more thoroughly and by computing finite-temperature free energies. Both would require considerable further investment of computational resources, however.

The ROY landscapes presented here suggest that the Z′ = 1 polymorphs and, with a lesser degree of confidence, the Z′ = 2 polymorphs with the most stable lattice energies have already been discovered. Further polymorph discoveries at ambient pressure would seem to require experimental ingenuity to trap metastable forms, similar to the efforts involved in crystallizing forms R18, Y19, and Y04. On the other hand, there may be potential for discovering a new polymorph at higher pressure. The YT04 and PO13 polymorphs are predicted to become more stable than form Y in the ∼1–10 GPa range. Around 10 GPa, however, a new, unknown candidate structure is predicted to become enthalpically more stable than all experimentally-known polymorphs. Given the structural similarities of this candidate to form Y, perhaps this new structure might be realized experimentally by subjecting form Y to pressures around 10 GPa. Alternatively, liquid recrystallizations at high pressures could also be considered.

Finally, the work here demonstrates how conformational energy corrections can be applied to entire crystal energy landscapes to improve their accuracy considerably. Given the small computational cost of these corrections—only a few percent of the time spent on DFT structure optimizations—they should be considered whenever conformational polymorphs exhibit varying degrees of intramolecular π conjugation or any other features that are likely to manifest in significant delocalization error.

## Author contributions

The Imperial College team performed the initial stages of the crystal structure prediction. G. J. O. B. performed the subsequent quantum mechanical refinement, with help from C. G., and analyzed the data. G. J. O. B. wrote the paper with input from all other co-authors.

## Conflicts of interest

There are no conflicts to declare.

## Supplementary Material

SC-013-D1SC06074K-s001

SC-013-D1SC06074K-s002
