# Supplementary material for: How many more polymorphs of ROY remain undiscovered
Source: Chem Sci. 2021 Dec 13;13(5):1288–97. doi: 10.1039/d1sc06074k (PMC8809489; doi:10.1039/d1sc06074k)
Supplement: SC-013-D1SC06074K-s002 [file SC-013-D1SC06074K-s002.pdf]

Electronic Supplementary Material (ESI) for Chemical Science.
This journal is © The Royal Society of Chemistry 2022

Supporting Information for "How many more polymorphs of ROY remain undiscovered?"

Gregory J. O. Beran,[*,†] Isaac J. Sugden,[‡] Chandler Greenwell,[†] David H. Bowskill,[‡] Constantinos C. Pantelides,[‡] and Claire S. Adjiman[‡]

†*Department of Chemistry, University of California Riverside, Riverside CA 92521, USA*

‡*Department of Chemical Engineering, Centre for Process Systems Engineering, Imperial College London, London, SW7 2AZ, United Kingdom*

E-mail: gregory.beran@ucr.edu

Phone: +1 (951) 827-7869

Contents

S1 Crystal structure prediction methods **2**

S2 Additional Data **6**

S2.1 Comparison of DFT and experimental crystal structures 6

S2.2 ROY conformational energy profile 7

S2.3 Relative energies of the ROY polymorphs 8

S2.4 Analysis of CSP structures with $Z' = 2$ 14

S2.5 High-pressure calculations 16

References **17**

S1 Crystal structure prediction methods

Generation of putative crystal structures was undertaken through six stages: (i) isolated molecule studies; (ii) global search; (iii) refinement with a transferable repulsion/dispersion (r/d) potential; (iv) refinement with a tailored r/d potential; (v) structure geometry refinement with B86bPBE-XDM, and (vi) final single-point energies with B86bPBE-XDM + ΔSCS-MP2D. The first three stages follow the general strategy presented in Pantelides et al.[1] and were employed in previous work on ROY.[2] However, the structure prediction process here was performed from scratch using the latest version of the codes and methodology as well as a consistent B3LYP level of theory throughout (instead of the mixed set of methods used in the earlier study). In particular, the version of CrystalPredictor used here includes an improved surrogate model (Local Approximate Model or LAM) that offers greater accuracy and speed through the use of adaptive LAM point generation[3] and LAM smoothing.[4] These stages are described in greater detail below.

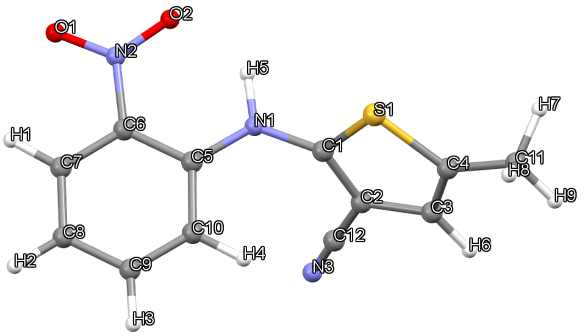

Figure S1: Atomic labeling for ROY corresponding to the discussion below.

Stage I: Isolated molecule studies. The gas-phase molecular geometry of ROY was first optimized at the B3LYP/6-31G(d,p) level of theory using Gaussian 09.[5] Based on the insight gained from a previous ROY study,[2] two conformational degrees of freedom were chosen as independent variables for the purpose of lattice energy minimization at the global search stage, namely C6-C5-N1-C1 (θ_1) and C5-N1-C1-C2 (θ_2) (see Figure S1). Their feasible ranges were determined by selecting values for which the increase in intramolecular energy over that of the *in vacuo* conformation is lower than 20 kJ/mol in one-dimensional scans, giving $\theta_1 \in [-20.0, 180.0]$ and $\theta_2 \in [100.0, 260.0]$. The adaptive Local Approximate Model (LAM) algorithm[3] was used to generate a database of 38 LAMs that describe the conformationally-dependent properties within this space, using an initial uniform grid generated with $\Delta\theta = \pm 20°$. The high-energy ($\Delta^{\star}$) and accuracy cutoff ($\Delta^{\star\star}$) parameters were set to 20 kJ/mol and 1.0 kJ/mol, respectively.

Stage II: Global crystal structure search. The global search for low-energy minima on the Z'=1 lattice energy surface was performed using CrystalPredictor II.[6] The smoothed intramolecular potential algorithm[4] was employed to describe intramolecular interactions

and point charges for electrostatic interactions, while r/d parameters were taken from the FIT potential set[7–10] Two million minimizations in 61 space groups were carried out. Structures within 20 kJ/mol of the global minimum were clustered using the CSD Python API[11] to compute $RMSD_{15}$ values.[12] If two structures are found to have an $RMSD_{15}$ less than 0.3 Å, a lattice energy difference of less than 0.2 kJ/mol and a density difference of less than 1.0 kg/m^3, the higher-energy structure is eliminated. This led to the identification of 2,869 distinct structures within 20 kJ/mol of the global minimum.

Stage III: Crystal structure refinement with a transferable r/d potential: CrystalOptimizer[13] was used to perform local minimizations of the lowest 1,000 structures determined at Stage II, at the B3LYP/6-31G(d,p) level of theory, with additional flexibility introduced (torsional angles C10-C5-N1-C1, O1-N2-C6-C5, O2-N2-C6-C5, H5-N1-C1-C2 and bond angles N1-C1-C2, C5-N1-C1, C10-C5-N1, O1-N2-C6, O2-N2-C6, H5-N1-C1). Electrostatic interactions were described using multipoles (up to hexadecapoles) while r/d potential parameters were taken from the FIT potential set.[7–10]

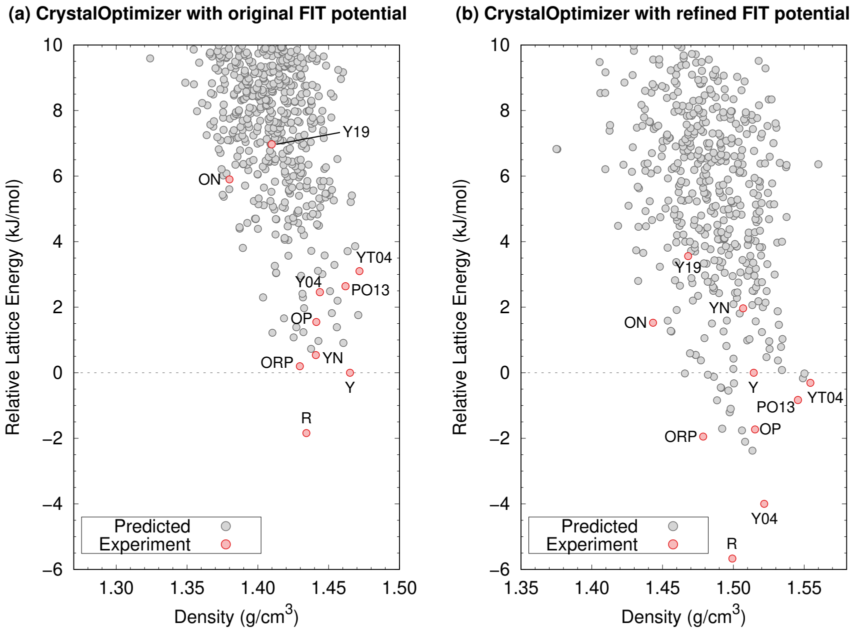

Figure S2: Comparison of the CSP landscapes before and after refinement of the FIT potential.

Stage IV: Further crystal structure refinement with a tailored r/d potential: The 50 lowest-energy structures from Stage III were fully relaxed with dispersion-corrected periodic planewave DFT calculation in QuantumEspresso[14,15] using the B86bPBE functional[16,17] and the exchange-dipole moment (XDM) dispersion correction.[18] This provided a set of optimized crystal geometries and energies which were then used as reference data for fitting an updated set of r/d potential parameters that are tailored to the ROY molecule. The CrystalEstimator[19] code was used for this purpose. The updated r/d parameter estimates, listed in Table 1, were then used to re-refine the lowest 1,000 structures from the global search at Stage II, using the same conformational degrees of freedom and electrostatic

model as at Stage III.

The final set of ∼300 structures carried forward represents the union of all structures which lie within 10 kJ/mol of the global minimum on either the Stage III or Stage IV landscapes, as indicated in Figure 2a of the main paper. This set was augmented with the structures of the three experimental polymorphs which have Z'=2: R05, R18, and RPL.

Table S1: Tailored Buckingham potential parameters for ROY at the B3LYP/6-31G(d,p) level of theory. The label "Hp" denotes hydrogen atoms bonded to polar electronegative atoms (O or N).

Atom 1	Atom2	A (eV)	B (Å)	C (eV Å^6)
C	C	2676.902	0.277777778	20.6113
C	H	290.8399	0.272479564	2.8769
C	N	3084.705	0.27100271	18.4061
C	O	3886.035	0.264550265	22.7715
C	Hp	1984.116	0.242130751	0.0
C	S	2534.239	0.289826	14.736
H	H	38.4716	0.267379679	0.4447
H	N	385.1455	0.265957447	5.658
H	O	378.3938	0.25974026	4.9875
H	Hp	33.3432	0.238095238	0.0
H	S	757.0927	0.284063	16.1177
N	N	2796.711	0.264550265	38.0875
N	O	2112.153	0.258397933	2.7457
N	Hp	395.0279	0.236966825	0.0
N	S	2891.83	0.282458	37.1543
O	O	4753.345	0.252525253	32.9007
O	Hp	175.5761	0.232018562	0.0
O	S	3042.481	0.275455	21.326
Hp	Hp	62607.3	0.214592275	0.0
Hp	S	2235.112	0.251234	0.0
S	S	4086.27	0.302966	40.0768

Stage V: DFT relaxation of the crystal structures.

All ∼300 structures carried forward from Stages III and IV were fully relaxed with B86bPBE-XDM using Quantum Espresso. After removal of duplicate structures which coalesced in the DFT optimizations, 264 unique structures remained.

The B86bPBE-XDM calculations employed a 50 Ry planewave energy cutoff and reciprocal space Monkhorst-Pack k-point grid densities of 0.04 Å^{-1} or better. Core electrons were treated with the projector augmented wave (PAW) approach using potentials produced with Atomic v6.1.[14] The gas-phase DFT energies for the monomer conformational energy correction were computed with the same parameters in a periodic box with an intermolecular spacing of at least 20 Å between any two periodic image atoms. Due to the large unit cell size, only the Γ-point was sampled in reciprocal space. The high-pressure calculations relaxed the structures under isotropic hydrostatic pressures ranging from 1–10 GPa.

Stage VI: Conformational energy correction and final energy ranking.
Finally, given the known problems GGA (and many other) density functionals have describing the ROY conformations, the final landscape was obtained by applying a single-point monomer conformational energy correction at the SCS-MP2D level of theory to each crystal from Stage V. The corrected energy per unit cell is computed as,

$$E_{crystal}^{corrected} = E_{crystal}^{DFT} + \sum_{i} \left(E_{mon,i}^{SCS-MP2D} - E_{mon,i}^{DFT} \right) \tag{1}$$

Energies per molecule are obtained by dividing by Z. The final results are denoted as B86bPBE-XDM + ΔSCS-MP2D. Here, the final energies were evaluated relative to that of form Y.

The SCS-MP2D calculations were performed using PSI4[20] (for MP2) and a custom implementation of the spin-component scaling and dispersion correction.[21] The SCS-MP2D energy at the complete-basis-set (CBS) limit is computed as the HF/aug-cc-pVQZ energy plus the scaled same-spin and opposite-spin MP2/CBS correlation energies. We then subtract the correspondingly scaled amounts of same- and opposite-spin uncoupled Hartree-Fock (UCHF) dispersion energies and replace them with the full, unscaled coupled Kohn-Sham (CKS) dispersion energy. See Ref 22 for details of these corrections and the values of the c_{os} and c_{ss} coefficients.

$$E_{CBS}^{SCS\text{-}MP2D} = E_{aQZ}^{HF} + c_{os} E_{CBS}^{MP2,os} + c_{ss} E_{CBS}^{MP2,ss} - c_{os} E_{UCHF}^{disp,os} - c_{ss} E_{UCHF}^{disp,ss} + E_{CKS}^{disp,tot} \tag{2}$$

The complete-basis-set (CBS)-limit same-spin and opposite-spin correlation energies were extrapolated[23] from density-fitted MP2 calculations performed in the aug-cc-pVTZ and aug-cc-pVQZ basis sets:

$$E_{CBS}^{MP2,ss} = \frac{4^3 E_{aQZ}^{MP2,ss} - 3^3 E_{aTZ}^{MP2,ss}}{4^3 - 3^3} \qquad E_{CBS}^{MP2,os} = \frac{4^3 E_{aQZ}^{MP2,os} - 3^3 E_{aTZ}^{MP2,os}}{4^3 - 3^3} \tag{3}$$

S2 Additional Data

S2.1 Comparison of DFT and experimental crystal structures

Table S2 compares the DFT-optimized crystal structures against experimental ones reported in the Cambridge Structure Database in terms of densities and structure overlays. Fifteen-molecule structure overlays were used for the comparison ($RMSD_{15}$ metric), as implemented in Mercury. The densities of the structures optimized with B86bPBE-XDM are consistently higher than experimental ones due to the neglect of zero-point vibrational energy and the thermal expansion of the crystals at finite temperatures. Most of the experimental structures were determined at room temperature, though a few were taken at lower temperatures, as indicated in the Table. The role of thermal expansion can be seen in the fact that the density differences between the experimental and DFT crystal structures are about twice as large for the polymorphs whose experimental structures were solved at room temperature compared to those solved at 100–150 K.

Table S2: Comparison of experimental and DFT-optimized crystal structures of ROY based on density and the $rmsd_{15}$ metric. The CSD RefCodes and experimental temperatures are provided.

			Density (g/cm^3)		
Polymorph	CSD RefCode	Temperature	Expt.	DFT	$rmsd_{15}$ (Å)
Y	QAXMEH22	Room Temp	1.448	1.537	0.221 Å
YT04	QAXMEH12	Room Temp	1.473	1.557	0.224 Å
R	QAXMEH02	Room Temp	1.438	1.520	0.166 Å
OP	QAXMEH03	Room Temp	1.435	1.539	0.204 Å
YN	QAXMEH04	Room Temp	1.431	1.518	0.238 Å
Y04	QAXMEH53	100 K	1.493	1.539	0.168 Å
R05	QAXMEH31	250 K	1.434	1.522	0.206 Å
PO13	QAXMEH52	Room Temp	1.458	1.569	0.259 Å
ON	QAXMEH	Room Temp	1.428	1.521	0.221 Å
ORP	QAXMEH05	Room Temp	1.429	1.508	0.198 Å
R18	QAXMEH57	150 K	1.480	1.521	0.068 Å
Y19	QAXMEH60	100 K	1.470	1.510	0.214 Å
				**Mean +/- Std Dev:**	**0.225 ± 0.079 Å**

S2.2 ROY conformational energy profile

Figure S3 plots the key gas-phase conformational energy profile for ROY. It shows how a GGA functional like B86bPBE-XDM over-stabilizes the more planar conformations, including those associated with red and orange polymorphs, relative to those less-planar conformations associated with Y polymorphs. It exhibits a root-mean-square (rms) error of 2.5 kJ/mol relative to complete-basis-set CCSD(T) benchmarks.[24] In contrast, complete-basis-set SCS-MP2D performs much better, with an rms error of 0.5 kJ/mol. See earlier studies for more examples of how DFT delocalization error impacts the conformational energies.[24–27]

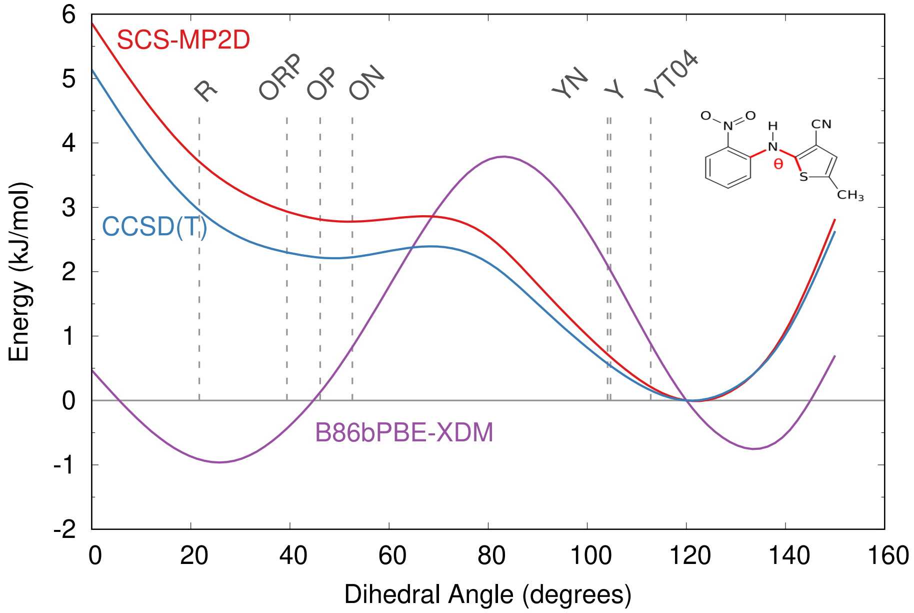

Figure S3: Conformational energy profile for rotating the key S-C-N-C dihedral angle θ. The experimental dihedral angles associated with several ROY polymorphs are indicated.

S2.3 Relative energies of the ROY polymorphs

Table S3 lists the computed lattice energies and energies relative to form Y as computed with B86bPBE-XDM and after the SCS-MP2D monomer correction is applied. These relative energies correspond to the crystal energy landscapes presented in the main paper. The experimental polymorphs are indicated in bold, while the high-pressure candidate structure #15 is highlighted in red. A CIF file containing all crystal structures is provided separately.

Table S3: Computed lattice and relative ROY polymorph energies as computed with B86bPBE-XDM before and after the SCS-MP2D monomer conformational energy correction is applied. Relative energies are computed relative to form Y.

Structure Rank	Polymorph	Space Group	Density (g/cm^3)	B86bPBE-XDM E_{latt} (kJ/mol)	B86bPBE-XDM E_{rel} (kJ/mol)	SCS-MP2D-corrected E_{latt} (kJ/mol)	SCS-MP2D-corrected E_{rel} (kJ/mol)
**0001**	**Y**	$P21/n$	**1.5366**	**-123.16**	**0.000**	**-128.25**	**0.000**
**0002**	**YT04**	$P21/n$	**1.5573**	**-123.60**	**-0.440**	**-127.61**	**0.642**
**0003**	**R**	$P\bar{1}$	**1.5196**	**-129.55**	**-6.388**	**-127.42**	**0.825**
**0004**	**OP**	$P21/n$	**1.5392**	**-126.96**	**-3.802**	**-126.64**	**1.607**
**0005**	**YN**	$P\bar{1}$	**1.5183**	**-122.23**	**0.930**	**-125.94**	**2.313**
**0006**	**Y04**	$P\bar{1}$	**1.5386**	**-124.90**	**-1.740**	**-125.83**	**2.417**
**0007**	**R05**	$P2_1$	**1.5222**	**-129.64**	**-6.479**	**-125.64**	**2.607**
0008		$Pbca$	1.5038	-123.09	0.069	-125.57	2.682
0009		$P\bar{1}$	1.5330	-122.40	0.761	-125.54	2.706
**0010**	**PO13**	$P21/c$	**1.5686**	**-127.64**	**-4.474**	**-125.45**	**2.799**
0011		$P21/c$	1.5307	-121.91	1.251	-125.28	2.972
**0012**	**ON**	$P21/c$	**1.5208**	**-127.44**	**-4.280**	**-125.23**	**3.016**
0013		$P\bar{1}$	1.5247	-126.55	-3.392	-125.20	3.045
0014		$P21/c$	1.5376	-127.97	-4.808	-124.86	3.392
**0015**	**High Press.**	$P21/n$	**1.5502**	**-120.75**	**2.413**	**-124.60**	**3.644**
0016		$P2_12_12_1$	1.5045	-120.57	2.595	-124.50	3.747
0017		$P2_12_12_1$	1.5449	-130.88	-7.721	-124.46	3.785
0018		$C2/c$	1.5404	-126.67	-3.507	-124.12	4.124
0019		$P21/c$	1.5226	-127.87	-4.709	-124.08	4.167
0020		$P21/n$	1.4907	-121.81	1.349	-124.01	4.239
0021		$P21/c$	1.5430	-126.78	-3.613	-124.01	4.241
0022		$P21/c$	1.5541	-126.70	-3.534	-123.99	4.258
**0023**	**ORP**	$Pbca$	**1.5081**	**-127.85**	**-4.684**	**-123.96**	**4.290**
0024		$P21/c$	1.5176	-124.48	-1.314	-123.88	4.371
0025		$P\bar{1}$	1.5689	-127.27	-4.108	-123.79	4.460
0026		$P21/c$	1.5269	-119.72	3.446	-123.78	4.469
0027		$P21/c$	1.5169	-119.67	3.492	-123.73	4.516
0028		$P21/n$	1.5479	-129.91	-6.751	-123.66	4.593
0029		$P21/c$	1.5638	-125.70	-2.541	-123.53	4.723
0030		$P21/n$	1.5642	-126.97	-3.806	-123.50	4.749
0031		$C2/c$	1.5197	-124.39	-1.227	-123.50	4.751
**0032**	**R18**	$P\bar{1}$	**1.5207**	**-130.03**	**-6.871**	**-123.39**	**4.860**
0033		$P21/c$	1.5199	-125.96	-2.795	-123.38	4.868
0034		$P21/c$	1.4742	-119.23	3.931	-123.26	4.992
0035		$P21/c$	1.5572	-126.40	-3.239	-123.19	5.055
0036		$P21/c$	1.5193	-124.60	-1.439	-123.11	5.142
**0037**	**RPL**	$Pbca$	**1.5026**	**-127.14**	**-3.979**	**-123.09**	**5.160**

Continued on next page

Continued from previous page

Structure Rank	Polymorph	Space Group	Density (g/cm^3)	B86bPBE-XDM E_{latt} (kJ/mol)	B86bPBE-XDM E_{rel} (kJ/mol)	SCS-MP2D-corrected E_{latt} (kJ/mol)	SCS-MP2D-corrected E_{rel} (kJ/mol)
0038		$P21/c$	1.5015	-118.89	4.276	-123.01	5.236
0039		$Fdd2$	1.5349	-124.06	-0.894	-123.00	5.252
0040		$P2_12_12_1$	1.4867	-125.38	-2.214	-122.98	5.269
0041		$P21/c$	1.5464	-123.72	-0.557	-122.94	5.309
0042		$P21/n$	1.5260	-119.00	4.163	-122.81	5.436
0043		$P21/n$	1.4976	-128.84	-5.678	-122.78	5.465
0044		$P21/c$	1.5129	-125.89	-2.724	-122.64	5.610
0045		$P2_12_12_1$	1.5277	-125.75	-2.584	-122.63	5.615
0046		$P21/c$	1.5372	-126.60	-3.434	-122.60	5.650
0047		$P21/c$	1.5057	-127.00	-3.834	-122.59	5.658
0048		$P\bar{1}$	1.5271	-122.71	0.451	-122.56	5.693
0049		$P21/c$	1.5071	-122.91	0.253	-122.51	5.737
0050		$P\bar{1}$	1.5283	-122.46	0.698	-122.50	5.753
0051		$I2$	1.5055	-122.02	1.142	-122.48	5.766
0052		$P21/n$	1.4859	-120.95	2.209	-122.46	5.791
0053		$P\bar{1}$	1.4957	-123.35	-0.185	-122.45	5.800
0054		$P2_12_12$	1.5349	-124.41	-1.244	-122.45	5.802
0055		$Pbca$	1.4847	-120.54	2.619	-122.42	5.829
0056		$P21/c$	1.5171	-124.80	-1.635	-122.28	5.965
0057		$P21/n$	1.5252	-128.06	-4.899	-122.24	6.007
0058		$Pbcn$	1.5427	-122.48	0.685	-122.21	6.042
0059		$P\bar{1}$	1.5098	-120.79	2.376	-122.17	6.075
0060		$P21/n$	1.4904	-123.98	-0.815	-122.16	6.088
0061		$Pbca$	1.4961	-119.53	3.637	-122.12	6.132
0062		$P21/n$	1.4961	-119.40	3.763	-122.08	6.168
0063		$P21/c$	1.5226	-122.31	0.848	-122.07	6.182
0064		$P21/n$	1.5152	-120.00	3.162	-122.01	6.238
0065		$P21/c$	1.5216	-122.53	0.635	-121.96	6.286
0066		$P21/c$	1.5317	-125.41	-2.249	-121.93	6.322
0067		$P21/c$	1.4905	-118.53	4.637	-121.75	6.497
0068		$P\bar{1}$	1.5377	-127.01	-3.849	-121.75	6.501
0069		$P21/n$	1.5390	-127.25	-4.092	-121.73	6.516
0070		$P\bar{1}$	1.5220	-126.72	-3.559	-121.66	6.591
0071		$C2/c$	1.4921	-120.63	2.529	-121.64	6.604
**0072**	**Y19**	$P21/c$	**1.5104**	**-122.66**	**0.504**	**-121.64**	**6.606**
0073		$P21/c$	1.5174	-127.81	-4.650	-121.59	6.654
0074		$P21/n$	1.4931	-117.89	5.268	-121.59	6.662
0075		$Aba2$	1.5005	-116.70	6.461	-121.51	6.736
0076		$C2/c$	1.5165	-118.75	4.415	-121.50	6.745
0077		$I2/c$	1.5538	-124.96	-1.801	-121.45	6.797
0078		$C2/c$	1.5401	-124.13	-0.972	-121.44	6.805
0079		$Fdd2$	1.5344	-123.07	0.097	-121.43	6.815
0080		Pn	1.5207	-118.29	4.873	-121.42	6.829
0081		$P21/n$	1.4809	-119.57	3.596	-121.40	6.853
0082		$P21/c$	1.5042	-118.61	4.549	-121.38	6.871
0083		$P21/c$	1.5083	-121.31	1.857	-121.35	6.895
0084		$Pccn$	1.5169	-127.56	-4.399	-121.35	6.901
0085		$C2/c$	1.5006	-125.97	-2.809	-121.33	6.916
0086		$Pbca$	1.5119	-125.32	-2.158	-121.33	6.919

Continued on next page

Continued from previous page

Structure Rank	Polymorph	Space Group	Density (g/cm^3)	B86bPBE-XDM E_{latt} (kJ/mol)	B86bPBE-XDM E_{rel} (kJ/mol)	SCS-MP2D-corrected E_{latt} (kJ/mol)	SCS-MP2D-corrected E_{rel} (kJ/mol)
0087		$P\bar{1}$	1.5243	-127.28	-4.119	-121.29	6.955
0088		$I2/c$	1.5041	-121.62	1.543	-121.29	6.957
0089		$P21/c$	1.5155	-125.29	-2.129	-121.29	6.958
0090		$P\bar{1}$	1.4904	-118.90	4.266	-121.26	6.986
0091		$P21/n$	1.5260	-117.32	5.840	-121.26	6.990
0092		$Pbcn$	1.5091	-119.86	3.300	-121.25	6.995
0093		$P21/c$	1.4947	-116.19	6.971	-121.21	7.038
0094		$P21/n$	1.5482	-123.97	-0.806	-121.14	7.110
0095		$Fdd2$	1.5076	-124.53	-1.368	-121.10	7.147
0096		$P21/c$	1.5054	-118.86	4.306	-121.10	7.153
0097		$P\bar{1}$	1.4983	-121.03	2.128	-121.09	7.162
0098		$P21/n$	1.4855	-118.07	5.092	-121.05	7.201
0099		$P21/n$	1.4890	-118.12	5.045	-121.02	7.231
0100		$P21/n$	1.5220	-126.93	-3.764	-120.97	7.279
0101		$Pca2_1$	1.5263	-122.96	0.201	-120.89	7.362
0102		$P21/c$	1.4791	-117.58	5.582	-120.76	7.492
0103		Cc	1.4837	-124.13	-0.970	-120.72	7.532
0104		$P21/c$	1.5023	-117.97	5.194	-120.67	7.579
0105		$P21/c$	1.4932	-118.23	4.935	-120.66	7.586
0106		$P21/c$	1.5138	-122.52	0.642	-120.64	7.605
0107		$Fdd2$	1.5210	-123.41	-0.244	-120.64	7.609
0108		$P21/c$	1.4979	-125.27	-2.106	-120.62	7.627
0109		$P21/c$	1.4762	-118.37	4.792	-120.48	7.765
0110		$P21/c$	1.5228	-118.22	4.946	-120.46	7.784
0111		$Pbca$	1.4655	-122.25	0.912	-120.46	7.790
0112		$C2/c$	1.5173	-126.80	-3.639	-120.45	7.803
0113		$C2/c$	1.5299	-125.89	-2.727	-120.44	7.813
0114		$Pna2_1$	1.4819	-122.60	0.559	-120.40	7.853
0115		$P21/c$	1.5432	-122.60	0.558	-120.35	7.900
0116		$P21/n$	1.5525	-123.44	-0.277	-120.30	7.948
0117		$Pna2_1$	1.4865	-119.54	3.627	-120.18	8.069
0118		$P\bar{1}$	1.5316	-126.86	-3.699	-120.18	8.069
0119		$P21/n$	1.4861	-119.09	4.072	-120.18	8.073
0120		$P21/c$	1.5348	-126.06	-2.894	-120.11	8.134
0121		$I2$	1.4854	-119.59	3.577	-120.10	8.152
0122		$Pccn$	1.5439	-123.94	-0.781	-120.09	8.163
0123		$P21/c$	1.5404	-123.82	-0.661	-120.08	8.166
0124		$Fdd2$	1.4726	-126.26	-3.095	-120.08	8.169
0125		$Pccn$	1.4883	-124.32	-1.157	-120.04	8.211
0126		$C2/c$	1.5269	-118.26	4.902	-120.03	8.216
0127		$P21/n$	1.5089	-120.63	2.536	-120.01	8.239
0128		$P21/n$	1.4878	-125.76	-2.601	-119.98	8.272
0129		$P\bar{1}$	1.5334	-126.97	-3.803	-119.96	8.285
0130		$Pbcn$	1.5065	-120.91	2.251	-119.95	8.295
0131		$P2_12_12_1$	1.5131	-124.72	-1.561	-119.79	8.459
0132		$P\bar{1}$	1.4960	-125.71	-2.543	-119.78	8.468
0133		$P21/n$	1.4950	-126.06	-2.893	-119.72	8.527
0134		$Pbcn$	1.4746	-119.68	3.478	-119.69	8.556
0135		$P21/c$	1.5111	-118.36	4.800	-119.67	8.578

Continued on next page

Continued from previous page

Structure Rank	Polymorph	Space Group	Density (g/cm^3)	B86bPBE-XDM E_{latt} (kJ/mol)	B86bPBE-XDM E_{rel} (kJ/mol)	SCS-MP2D-corrected E_{latt} (kJ/mol)	SCS-MP2D-corrected E_{rel} (kJ/mol)
0136		$P21/n$	1.4961	-118.10	5.063	-119.65	8.595
0137		$P21/n$	1.5118	-126.46	-3.293	-119.54	8.704
0138		$P\bar{1}$	1.5002	-124.25	-1.090	-119.53	8.721
0139		$P\bar{1}$	1.5304	-118.76	4.403	-119.49	8.760
0140		$P21/n$	1.4909	-125.12	-1.953	-119.48	8.771
0141		$I2/a$	1.5541	-122.82	0.343	-119.45	8.801
0142		$Pnna$	1.4681	-121.11	2.057	-119.43	8.819
0143		$P2_12_12_1$	1.5372	-123.27	-0.107	-119.42	8.831
0144		$P21/c$	1.4995	-123.95	-0.786	-119.39	8.860
0145		$I2$	1.5294	-120.98	2.185	-119.34	8.909
0146		$P\bar{1}$	1.4797	-118.96	4.203	-119.32	8.925
0147		$I2/c$	1.4766	-118.60	4.567	-119.31	8.938
0148		$P2_1$	1.5029	-122.70	0.465	-119.31	8.943
0149		$Pbca$	1.4654	-122.07	1.095	-119.25	8.995
0150		$C2/c$	1.5322	-122.47	0.694	-119.24	9.006
0151		$Pna2_1$	1.4942	-121.52	1.640	-119.21	9.041
0152		$P21/c$	1.5274	-118.11	5.053	-119.21	9.043
0153		$Pna2_1$	1.5259	-124.56	-1.402	-119.16	9.084
0154		$P21/n$	1.5169	-121.31	1.851	-119.08	9.173
0155		$C2/c$	1.5075	-125.38	-2.213	-119.00	9.246
0156		$C2/c$	1.4988	-118.82	4.346	-118.98	9.269
0157		Pc	1.5065	-117.45	5.711	-118.95	9.299
0158		$Fdd2$	1.5407	-121.84	1.321	-118.91	9.335
0159		$Pbca$	1.4813	-120.50	2.667	-118.89	9.357
0160		$P21/c$	1.4797	-116.51	6.652	-118.83	9.418
0161		$P21/n$	1.5043	-116.99	6.171	-118.79	9.463
0162		$C2/c$	1.5440	-122.18	0.987	-118.78	9.470
0163		$Pbca$	1.4591	-124.74	-1.575	-118.77	9.474
0164		$P2_1$	1.5033	-116.96	6.204	-118.68	9.570
0165		$P\bar{1}$	1.5088	-124.98	-1.820	-118.66	9.592
0166		$P21/c$	1.5040	-117.92	5.244	-118.56	9.688
0167		$P21/c$	1.4924	-122.02	1.141	-118.52	9.732
0168		$C2/c$	1.5501	-120.82	2.338	-118.44	9.813
0169		$P2/n$	1.4721	-116.92	6.240	-118.43	9.816
0170		$P21/c$	1.4795	-117.55	5.609	-118.42	9.825
0171		$Pbca$	1.5259	-124.17	-1.011	-118.39	9.859
0172		$C2/c$	1.4828	-114.59	8.574	-118.33	9.917
0173		Cc	1.5140	-115.58	7.582	-118.27	9.979
0174		$P\bar{1}$	1.4888	-124.46	-1.294	-118.26	9.991
0175		$Pbcn$	1.4911	-113.44	9.718	-118.22	10.029
0176		$P21/n$	1.5517	-121.77	1.394	-118.21	10.040
0177		$Fdd2$	1.4809	-122.89	0.276	-118.18	10.064
0178		$I2$	1.5327	-120.57	2.592	-118.18	10.072
0179		$P21/c$	1.5173	-114.43	8.734	-118.14	10.111
0180		$C2/c$	1.5143	-124.17	-1.012	-118.13	10.115
0181		$P21/c$	1.5041	-122.70	0.459	-118.10	10.149
0182		$P21/c$	1.4740	-117.44	5.719	-118.04	10.204
0183		$P21/n$	1.4750	-116.48	6.678	-118.03	10.215
0184		$P21/c$	1.4962	-117.21	5.948	-118.01	10.242

Continued on next page

Continued from previous page

Structure Rank	Polymorph	Space Group	Density (g/cm^3)	B86bPBE-XDM E_{latt} (kJ/mol)	E_{rel} (kJ/mol)	SCS-MP2D-corrected E_{latt} (kJ/mol)	E_{rel} (kJ/mol)
0185		$Pna2_1$	1.5419	-123.82	-0.656	-117.99	10.263
0186		$C2/c$	1.5195	-123.62	-0.458	-117.96	10.284
0187		$P21/n$	1.5062	-118.80	4.361	-117.95	10.303
0188		$P21/n$	1.5195	-119.30	3.866	-117.92	10.331
0189		$P21/c$	1.5243	-123.98	-0.815	-117.78	10.467
0190		$P21/c$	1.5189	-122.15	1.013	-117.76	10.487
0191		$C2/c$	1.4621	-120.78	2.381	-117.73	10.521
0192		$C2/c$	1.5415	-121.19	1.976	-117.70	10.551
0193		$P21/n$	1.4801	-117.91	5.250	-117.70	10.551
0194		$Pnna$	1.4668	-123.80	-0.641	-117.64	10.608
0195		$C2/c$	1.4900	-123.99	-0.831	-117.58	10.672
0196		$P21/c$	1.4669	-121.51	1.651	-117.55	10.694
0197		$P21/n$	1.5155	-116.71	6.452	-117.54	10.712
0198		$Fdd2$	1.5197	-117.54	5.623	-117.52	10.733
0199		$P21/n$	1.4841	-121.19	1.973	-117.51	10.737
0200		$P2/n$	1.5488	-120.24	2.921	-117.48	10.768
0201		$P2_12_12_1$	1.4844	-120.25	2.915	-117.47	10.783
0202		$Fdd2$	1.4791	-116.56	6.602	-117.46	10.789
0203		$P21/c$	1.4712	-117.93	5.232	-117.42	10.828
0204		$P21/c$	1.4710	-121.78	1.379	-117.42	10.832
0205		$C2/c$	1.4746	-118.16	5.003	-117.40	10.848
0206		$C2/c$	1.4800	-121.89	1.273	-117.35	10.899
0207		$I2/a$	1.4977	-117.41	5.755	-117.32	10.925
0208		$P21/c$	1.4977	-119.57	3.590	-117.31	10.938
0209		$P21/c$	1.5159	-124.72	-1.558	-117.27	10.975
0210		$P21/n$	1.5287	-119.24	3.922	-117.23	11.023
0211		$P2/n$	1.5362	-119.26	3.905	-117.22	11.025
0212		$P21/c$	1.4930	-120.89	2.275	-117.12	11.131
0213		$C2/c$	1.4658	-115.34	7.820	-117.06	11.190
0214		$P21/n$	1.5234	-123.43	-0.269	-116.97	11.278
0215		$P21/c$	1.4545	-120.62	2.540	-116.92	11.329
0216		$P21/c$	1.5038	-122.79	0.369	-116.83	11.419
0217		$P21/c$	1.4875	-122.34	0.826	-116.81	11.436
0218		$P2_12_12_1$	1.4968	-122.82	0.343	-116.80	11.445
0219		$I2/c$	1.4760	-113.60	9.558	-116.71	11.542
0220		$P21/n$	1.4795	-114.52	8.643	-116.66	11.588
0221		$P\bar{1}$	1.5202	-123.06	0.106	-116.63	11.623
0222		$P21/n$	1.5082	-123.35	-0.184	-116.61	11.638
0223		$I2/a$	1.5240	-114.81	8.352	-116.58	11.664
0224		$P21/c$	1.5285	-112.95	10.209	-116.47	11.775
0225		$C2/c$	1.5018	-120.89	2.271	-116.31	11.941
0226		$P21/c$	1.4405	-114.37	8.794	-116.26	11.985
0227		$Pbca$	1.5237	-123.20	-0.033	-116.07	12.180
0228		$P\bar{1}$	1.4752	-112.56	10.598	-116.03	12.215
0229		$P\bar{1}$	1.4747	-117.44	5.727	-115.96	12.286
0230		$Aba2$	1.5054	-122.33	0.828	-115.90	12.349
0231		$P21/c$	1.4808	-122.09	1.074	-115.72	12.532
0232		$C2/c$	1.5236	-117.72	5.441	-115.69	12.560
0233		$P21/n$	1.4770	-121.57	1.588	-115.61	12.634

Continued on next page

Continued from previous page

Structure Rank	Polymorph	Space Group	Density (g/cm^3)	B86bPBE-XDM E_{latt} (kJ/mol)	B86bPBE-XDM E_{rel} (kJ/mol)	SCS-MP2D-corrected E_{latt} (kJ/mol)	SCS-MP2D-corrected E_{rel} (kJ/mol)
0234		$Pna2_1$	1.4891	-120.61	2.550	-115.61	12.634
0235		$P\bar{1}$	1.5082	-115.38	7.780	-115.57	12.677
0236		$Pbca$	1.5108	-121.87	1.291	-115.56	12.692
0237		$P21/c$	1.4922	-121.90	1.265	-115.55	12.702
0238		$P21/c$	1.5105	-121.71	1.454	-115.49	12.757
0239		$Pbcn$	1.4897	-121.09	2.071	-115.39	12.860
0240		$I2/a$	1.5230	-121.28	1.884	-115.36	12.886
0241		$P2/n$	1.4628	-121.39	1.777	-115.31	12.943
0242		$P21/c$	1.5117	-111.31	11.857	-115.18	13.071
0243		$P21/n$	1.5270	-121.23	1.930	-115.11	13.138
0244		$P21/c$	1.4828	-120.82	2.338	-115.08	13.165
0245		$P21/n$	1.5030	-121.29	1.869	-114.97	13.275
0246		$Pbca$	1.5164	-114.09	9.072	-114.89	13.354
0247		$P21/c$	1.4897	-120.86	2.304	-114.84	13.409
0248		$P21/n$	1.4532	-111.15	12.016	-114.82	13.426
0249		$C2/c$	1.4773	-121.16	2.000	-114.72	13.530
0250		$P21/n$	1.5071	-121.75	1.417	-114.68	13.567
0251		$Pna2_1$	1.4566	-120.20	2.961	-114.46	13.784
0252		$C2/c$	1.5072	-119.93	3.236	-114.43	13.823
0253		$C2/c$	1.4840	-121.12	2.046	-114.38	13.873
0254		$P21/n$	1.4743	-112.56	10.606	-114.03	14.219
0255		$C2$	1.4752	-120.14	3.022	-113.96	14.286
0256		$Aba2$	1.5179	-112.64	10.522	-113.85	14.402
0257		$Pbca$	1.5211	-118.93	4.229	-113.72	14.524
0258		$Pbca$	1.4615	-110.24	12.920	-113.31	14.938
0259		Ic	1.4934	-118.25	4.916	-113.01	15.242
0260		$P21/c$	1.4635	-118.84	4.321	-112.43	15.817
0261		$Pbca$	1.4717	-109.29	13.869	-112.12	16.133
0262		$Pna2_1$	1.4632	-116.67	6.496	-111.66	16.592
0263		$C222_1$	1.4419	-117.22	5.944	-111.50	16.748
0264		$Pccn$	1.4381	-116.39	6.777	-110.74	17.512

S2.4 Analysis of CSP structures with $Z' = 2$

Nyman et al[26] performed a CSP study on ROY that searched over 19 space groups with Z'=2 and 38 space groups with Z'=1. This landscape was using dispersion-corrected PBE DFT calculations and a DFT-tailored force field using the GRACE program. The authors published both their final optimized predicted structures as well optimized geometries for each of the experimental forms that were known at the time. The search was imperfect—it found only six experimental polymorphs. It was also heavily biased toward red/orange forms due to delocalization error in the PBE functional; only 2% of the structures in the final set exhibit an SCNC dihedral angle of 90±40°, which is the approximate range associated with yellow polymorphs (as discussed in the main paper). In contrast, more than a third of the lower-energy structures reported in our search lie in that range.

Nevertheless, because our own search only examined Z'=1 structures, the Nyman et al landscape enables assessment of the importance of Z'=2 structures. We performed single-point B86bPBE-XDM energy calculations and applied the SCS-MP2D monomer energy correction to the 500 lowest-energy structures from their final landscape of 1,077 DFT-optimized structures. About three-quarters of these structures have Z'=2. Differences in the functionals and dispersion corrections between that study and our own leads to changes in crystal packing densities, intramolecular bond lengths, and overall energies that hinder direct comparison between the single-point energies for these structures and the energies from our own landscape. For example, the raw SCS-MP2D monomer energies for the experimental forms differ by up to 3.5 kJ/mol depending on which functional was used to optimize them. On the other hand, relative energies between forms are far more consistent across the two landscapes. Accordingly, Figure S4 presents the energies in each landscape relative to form Y from that same landscape, and the two landscapes are presented side-by-side.

Unsurprisingly, given the the PBE-NP results from Ref 26 and our B86bPBE-XDM data for our own landscape in the main paper, B86bPBE-XDM predicts the yellow forms on the Nyman et al landscape to be far less stable than both the red/orange experimental forms and most of the predicted structures. Performing the SCS-MP2D monomer correction transforms the landscape dramatically, and form Y becomes the most stable. As can be seen from Figure S4, the relative SCS-MP2D-corrected B86bPBE-XDM single-point energies of the experimental polymorphs are reasonably similar regardless of whether they are optimized with PBE-NP or B86bPBE-XDM. The energies of forms OP, ORP, PO13, R05, YN, and YT04 vs. Y vary by 0–0.3 kJ/mol between the two landscapes in Figures S4b and c. The exceptions are form ON, for which Nyman et al reported problems reproducing the experimental geometry with the PBE-NP model,[26] and form R, which is 0.9 kJ/mol less stable when optimized with PBE-NP.

Looking at the revised landscape after the conformational energy correction, we find few if any low-energy Z'=2 structures which compete with the low-energy experimental forms. R05 remains the lowest-energy Z'=2 structure. The next lowest predicted structure (Rank #19 in their list) with Z'=2 lies only slightly above R05 in energy. However, this structure involves two distinct *ab*-plane layers which alternate intramolecular conformation and packing motif along the *c* direction. One of these 2-D layers is very similar to the packing found in form YN, and experimental realization of this structure might be inhibited by preferential crystallization of the more stable YN polymorph. The other Z'=2 structures

lie in higher-energy regions of the landscape where many unobserved candidates lie and do not particularly stand out.

Overall this analysis of the CSP landscape from Ref 26 bolsters the argument in the main paper that the important low-energy polymorphs of ROY have already been found. It is worth reiterating, however, that this landscape is heavily biased toward more planar conformations of the ROY molecule, and the possibility of missing $Z' = 2$ structures with yellow-color conformations cannot currently be ruled out.

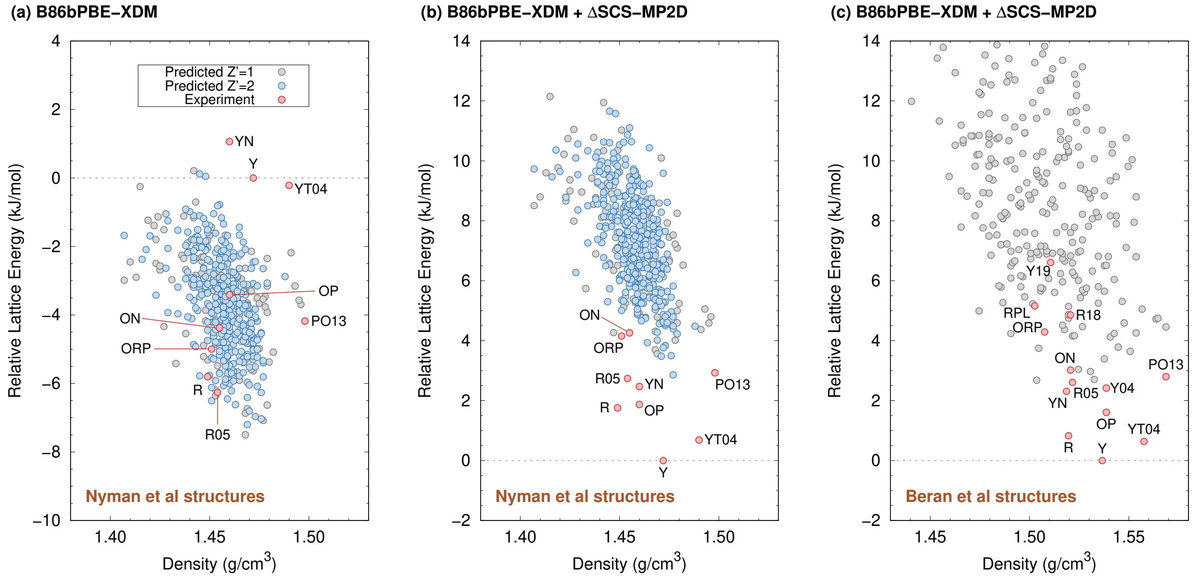

Figure S4: (a) The crystal energy landscape from Nyman et al,[26] after the single-point energy re-ranking with B86bPBE-XDM performed here. The landscape plotted here includes the 500 lowest-energy forms from their landscape plus the optimized experimental crystal structures they provided. (b) The Nyman et al landscape, after the SCS-MP2D monomer conformational energy correction is applied. (c) The final SCS-MP2D monomer-corrected landscape from Figure 2c of the main paper, which is repeated here for convenience.

S2.5 High-pressure calculations

Figure S5 highlights which structures from the B86bPBE-XDM + ΔSCS-MP2D monomer correction landscape were studied at higher pressures. The specific structures studied at high pressure are:

- ***Experimental:*** Y, YT04, R, OP, YN, Y04, R05, PO13, ON, ORP, R18, and Y19.
- ***CSP candidates:*** Ranks #9, #11, #13, #15, #17, #18, #21, #22, #25, #28, #29, #30, #35, and #41.

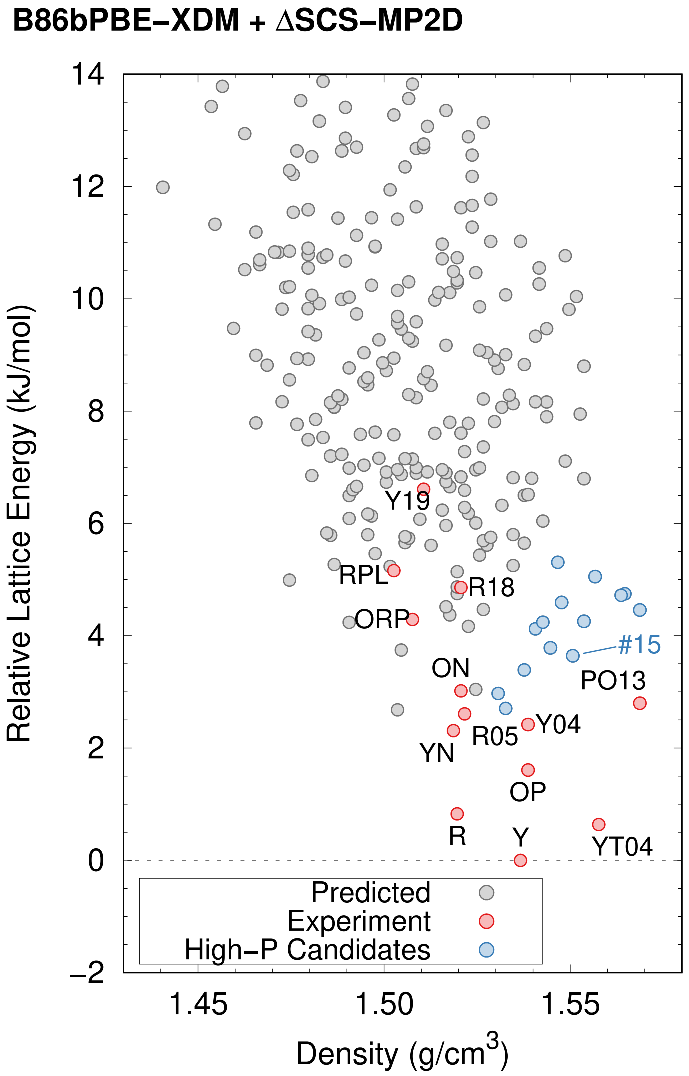

Figure S5: Pressure-dependent enthalpy calculations were performed for all experimental structures in red except RPL (Z=16) and for the candidate structures shown in blue. The rank #15 structure which becomes more stable than all experimental polymorphs around 10 GPa is indicated.

References

(1) Pantelides, C. C.; Adjiman, C. S.; Kazantsev, A. V. General Computational Algorithms for Ab Initio Crystal Structure Prediction for Organic Molecules. *Top. Curr. Chem.* **2014**, *345*, 25–58.

(2) Vasileiadis, M.; Kazantsev, A. V.; Karamertzanis, P. G.; Adjiman, C. S.; Pantelides, C. C. The polymorphs of ROY: application of a systematic crystal structure prediction technique. *Acta Cryst. B* **2012**, *68*, 677–685.

(3) Sugden, I.; Adjiman, C. S.; Pantelides, C. C. Accurate and efficient representation of intramolecular energy in ab initio generation of crystal structures. I. Adaptive local approximate models. *Acta Cryst. B* **2016**, *72*, 864–874.

(4) Sugden, I. J.; Adjiman, C. S.; Pantelides, C. C. Accurate and efficient representation of intramolecular energy in ab initio generation of crystal structures. II. Smoothed intramolecular potentials. *Acta Cryst. B* **2019**, *75*, 423–433.

(5) Frisch, M. J.; Trucks, G. W.; Schlegel, H. B.; Scuseria, G. E.; Robb, M. A.; Cheeseman, J. R.; Scalmani, G.; Barone, V.; Mennucci, B.; Petersson, G. A.; Nakatsuji, H.; Caricato, M.; Li, X.; Hratchian, H. P.; Izmaylov, A. F.; Bloino, J.; Zheng, G.; Sonnenberg, J. L.; Hada, M.; Ehara, M.; Toyota, K.; Fukuda, R.; Hasegawa, J.; Ishida, M.; Nakajima, T.; Honda, Y.; Kitao, O.; Nakai, H.; Vreven, T.; Montgomery, J. A., Jr.; Peralta, J. E.; Ogliaro, F.; Bearpark, M.; Heyd, J. J.; Brothers, E.; Kudin, K. N.; Staroverov, V. N.; Kobayashi, R.; Normand, J.; Raghavachari, K.; Rendell, A.; Burant, J. C.; Iyengar, S. S.; Tomasi, J.; Cossi, M.; Rega, N.; Millam, J. M.; Klene, M.; Knox, J. E.; Cross, J. B.; Bakken, V.; Adamo, C.; Jaramillo, J.; Gomperts, R.; Stratmann, R. E.; Yazyev, O.; Austin, A. J.; Cammi, R.; Pomelli, C.; Ochterski, J. W.; Martin, R. L.; Morokuma, K.; Zakrzewski, V. G.; Voth, G. A.; Salvador, P.; Dannenberg, J. J.; Dapprich, S.; Daniels, A. D.; Farkas, Ö.; Foresman, J. B.; Ortiz, J. V.; Cioslowski, J.; Fox, D. J. Gaussian 09 Revision E.01. 2009; Gaussian Inc. Wallingford CT.

(6) Habgood, M.; Sugden, I. J.; Kazantsev, A. V.; Adjiman, C. S.; Pantelides, C. C. Efficient Handling of Molecular Flexibility in Ab Initio Generation of Crystal Structures. *J. Chem. Theory Comput.* **2015**, 150306122603009.

(7) Cox, S. R.; Hsu, L.-Y.; Williams, D. E. Nonbonded potential function models for crystalline oxohydrocarbons. *Acta Cryst. A* **1981**, *37*, 293–301.

(8) Williams, D. E.; Cox, S. R. Nonbonded potentials for azahydrocarbons: the importance of the Coulombic interaction. *Acta Cryst. B* **1984**, *40*, 404–417.

(9) Coombes, D. S.; Price, S. L.; Willock, D. J.; Leslie, M. Role of electrostatic interactions in determining the crystal structures of polar organic molecules. A distributed multipole study. *J. Phys. Chem.* **1996**, *100*, 7352–7360.

(10) Beyer, T.; Price, S. L. Dimer or Catemer? Low-Energy Crystal Packings for Small Carboxylic Acids. *J. Phys. Chem. B* **2000**, *104*, 2647–2655.

(11) Groom, C. R.; Bruno, I. J.; Lightfoot, M. P.; Ward, S. C. The Cambridge Structural Database. *Acta Cryst. B* **2016**, *72*, 171–179.

(12) Chisholm, J. A.; Motherwell, W. D. S. COMPACK: A program for identifying crystal structure similarity using distances. *J. Appl. Crystall.* **2005**, *38*, 228–231.

(13) Kazantsev, A. V.; Karamertzanis, P. G.; Adjiman, C. S.; Pantelides, C. C. Efficient Handling of Molecular Flexibility in Lattice Energy Minimization of Organic Crystals. *J. Chem. Theory Comput.* **2011**, *7*, 1998–2016.

(14) Giannozzi, P.; Baroni, S.; Bonini, N.; Calandra, M.; Car, R.; Cavazzoni, C.; Ceresoli, D.; Chiarotti, G. L.; Cococcioni, M.; Dabo, I.; Dal Corso, A.; de Gironcoli, S.; Fabris, S.; Fratesi, G.; Gebauer, R.; Gerstmann, U.; Gougoussis, C.; Kokalj, A.; Lazzeri, M.; Martin-Samos, L.; Marzari, N.; Mauri, F.; Mazzarello, R.; Paolini, S.; Pasquarello, A.; Paulatto, L.; Sbraccia, C.; Scandolo, S.; Sclauzero, G.; Seitsonen, A. P.; Smogunov, A.; Umari, P.; Wentzcovitch, R. M. QUANTUM ESPRESSO: a modular and open-source software project for quantum simulations of materials. *J. Phys. Condens. Mat.* **2009**, *21*, 395502.

(15) Giannozzi, P.; Andreussi, O.; Brumme, T.; Bunau, O.; Buongiorno Nardelli, M.; Calandra, M.; Car, R.; Cavazzoni, C.; Ceresoli, D.; Cococcioni, M.; Colonna, N.; Carnimeo, I.; Dal Corso, A.; de Gironcoli, S.; Delugas, P.; DiStasio, R. A.; Ferretti, A.; Floris, A.; Fratesi, G.; Fugallo, G.; Gebauer, R.; Gerstmann, U.; Giustino, F.; Gorni, T.; Jia, J.; Kawamura, M.; Ko, H.-Y.; Kokalj, A.; Küçükbenli, E.; Lazzeri, M.; Marsili, M.; Marzari, N.; Mauri, F.; Nguyen, N. L.; Nguyen, H.-V.; Otero-de-la Roza, A.; Paulatto, L.; Poncé, S.; Rocca, D.; Sabatini, R.; Santra, B.; Schlipf, M.; Seitsonen, A. P.; Smogunov, A.; Timrov, I.; Thonhauser, T.; Umari, P.; Vast, N.; Wu, X.; Baroni, S. Advanced capabilities for materials modelling with Quantum ESPRESSO. *J. Phys. Condens. Mat.* **2017**, *29*, 465901.

(16) Becke, A. D. On the large-gradient behavior of the density functional exchange energy. *J. Chem. Phys.* **1986**, *38*, 7184–7187.

(17) Perdew, J. P.; Burke, K.; Ernzerhof, M. Generalized gradient approximation made simple. *Phys. Rev. Lett.* **1996**, *77*, 3865.

(18) Otero-de-la Roza, A.; Johnson, E. R. Van der Waals interactions in solids using the exchange-hole dipole moment model. *J. Chem. Phys.* **2012**, *136*, 174109.

(19) Bowskill, D. H.; Sugden, I. J.; Pantelides, C. C.; Adjiman, C. S. . *in preparation* **2021**,

(20) Parrish, R. M.; Burns, L. A.; Smith, D. G. A.; Simmonett, A. C.; DePrince, A. E.; Hohenstein, E. G.; Bozkaya, U.; Sokolov, A. Y.; Di Remigio, R.; Richard, R. M.; Gonthier, J. F.; James, A. M.; McAlexander, H. R.; Kumar, A.; Saitow, M.; Wang, X.; Pritchard, B. P.; Verma, P.; Schaefer, H. F.; Patkowski, K.; King, R. A.; Valeev, E. F.;

Evangelista, F. A.; Turney, J. M.; Crawford, T. D.; Sherrill, C. D. Psi4 1.1: An Open-Source Electronic Structure Program Emphasizing Automation, Advanced Libraries, and Interoperability. *J. Chem. Theory Comput.* **2017**, *13*, 3185–3197.

(21) The MP2D software can be downloaded at `https://github.com/Chandemonium/MP2D`.

(22) Greenwell, C.; Řezáč, J.; Beran, G. J. O. Spin-component-scaled and dispersion-corrected second-order Møller-Plesset perturbation theory: An alternative path toward chemical accuracy. *ChemRxiv* **2021**,

(23) Helgaker, T.; Klopper, W.; Koch, H.; Noga, J. Basis-set convergence of correlated calculations on water. *J. Chem. Phys.* **1997**, *106*, 9639–9646.

(24) Greenwell, C.; McKinley, J. L.; Zhang, P.; Zeng, Q.; Sun, G.; Li, B.; Wen, S.; Beran, G. J. O. Overcoming the difficulties of predicting conformational polymorph energetics in molecular crystals via correlated wavefunction methods. *Chem. Sci.* **2020**, *11*, 2200–2214.

(25) Thomas, S. P.; Spackman, M. A. The Polymorphs of ROY: A Computational Study of Lattice Energies and Conformational Energy Differences. *Austral. J. Chem.* **2018**, *71*, 279.

(26) Nyman, J.; Yu, L.; Reutzel-Edens, S. M. Accuracy and reproducibility in crystal structure prediction: the curious case of ROY. *CrystEngComm* **2019**, *21*, 2080–2088.

(27) Greenwell, C.; Beran, G. J. O. Inaccurate conformational energies still hinder crystal structure prediction in flexible organic molecules. *Cryst. Growth Des.* **2020**, *20*, 4875–4881.
